# Using technology to revolutionize cooperative learning: an opinion

**DOI:** 10.3389/fpsyg.2014.01156

**Published:** 2014-10-13

**Authors:** David W. Johnson, Roger T. Johnson

**Affiliations:** Educational Psychology, University of MinnesotaEdina, MN, USA

**Keywords:** cooperative learning, technology, social interdependence, cooperation, promotive interaction

## Nature of cooperative learning

Cooperative learning is an application of social interdependence theory (Deutsch, 1949, 1962; Johnson and Johnson, 1989, 2005, 2009). Social interdependence theory posits that there are two types of social interdependence, positive (cooperative) and negative (competitive). *Positive interdependence* (i.e., cooperation) exists when individuals perceive that they can reach their goals if and only if the other individuals with whom they are cooperatively linked also reach their goals. *Negative interdependence* (i.e., competition) exists when individuals perceive that they can obtain their goals if and only if the other individuals with whom they are competitively linked fail to obtain their goals. *No interdependence* (i.e., individualistic efforts) exists when individuals perceive that they can reach their goal regardless of whether other individuals in the situation attain or do not attain their goals. The basic premise of social interdependence theory is that the way in which interdependence is structured determines how individuals interact, and the interaction pattern determines the outcomes of the situation (Deutsch, 1949, 1962; Johnson and Johnson, 1989, 2005, 2009). Positive interdependence tends to result in promotive interaction (such as mutual help and assistance), negative interdependence tends to result in oppositional interaction (such as obstruction of each other's efforts), and no interdependence tends to result in the absence of interaction. Overall, positive goal interdependence and the resulting promotive interaction tends to result in greater efforts to achieve, more positive relationships, and greater psychological health than do negative or no goal interdependence. Detailed instructions in how to conduct cooperative learning lessons may be found in Johnson et al. (2013).

There is considerable evidence that in cooperative endeavors, face-to-face interactions are more effective than are on-line interactions (Johnson and Johnson, 2013). While the relative negative aspects of electronic interaction for cooperation are often pointed out, there may be too little discussion of how technology can facilitate and enhance cooperative endeavors. It is possible that technology may revolutionize how cooperative learning will function in the classrooms of the 21st Century. Technology may enhance the learning of basic skills such as reading, writing, and engaging in discussions. It may enhance the nature of reports and allow the covering of important events. It may change the nature of multimedia projects. Technology may expand communication and the way in which group members work together. It may structure inquiry projects, lead to each group establishing its own web page, and involve students in simulations. Finally, it may enable teachers to track the work of each student and each group and to manage courses and build learning communities more efficiently.

## Cooperative reading

Technology can facilitate the cooperative nature of reading the same or related material. Electronic devices such as the Kindle, Nook, and iPad (i.e., the Inkling application) allow members of a cooperative group to share with each other passages out of the books they are reading, highlight passages so others can note what one thought was important in the text material, and make notes for the other members of their group to read and respond to. Members of a cooperative group can even share passages from the books on Twitter and Facebook.

## Cooperative writing

Technology can facilitate learning how to write, improving the quality of one's writing, and working together in producing one document authored by the whole group. Google Docs may be used by cooperative groups to write or edit a joint document. A group of students can see and make changes to the joint document in real time, commenting on the document as a whole, or commenting on specific parts of the document. Group members can argue and disagree about the organization and wording of the document.

## Reflecting on a discussion

Technology can enrich and extend a discussion. Through the use of texting, Twitter, or social media sites such as Facebook, or even through a video conference, group members can comment on a discussion they had in class. Thus, discussions can continue indefinitely as students have the ability to communicate with each other at any time as they reflect on the previous discussion.

## Illustrating a report

Technology can improve a cooperative learning group's reports. Programs such as Flickr allow members of a cooperative group to upload photos and share them with the group, class, or the world. Each photo can have a comment stream to encourage discussion. If a group is preparing a presentation on Shakespeare, for example, photos can be gathered of his birthplace, houses he lived in, bars he hung out in, and so forth. Visual elements can then be added to any group report or product with the opportunity for viewers to comment on the visuals.

## Multimedia projects

One of the easiest blends of cooperative learning and technology is the assignment of multimedia projects. Technology may revolutionize the way in which cooperative learning groups work on such projects. The presentation can be a video, an animation, a slide show with music and a narration, or even a play or dance to music and narration. High quality multimedia projects require an understanding of the criteria and rubrics that will be used to assess the quality of the project, careful planning, and rehearsal. If the project includes a video, the group may wish to post it on YouTube. Thus, technology allows cooperative groups to combine a variety of medias to improve the quality of the project and its presentation.

## Covering relevant events

Technology enables members of a cooperative group to view events that relate to the material they are studying. Events relevant to a cooperative group's task or project may be covered through CoverItLive, which includes a moderated chat and live blog applications. Members of the group can publish comments, upload multimedia, embed photos, ask questions, and create newsflashes and scoreboards. Political speeches, presentations by other groups, world events, and even historical events can be covered and analyzed by a cooperative group.

## Communication and collaboration software

While initially he web was basically an electronic reference book, today there is a variety of software to facilitate communication and cooperation among group members. Instant messaging may be used for quick chats, blogs may be used for discussions, Google Sites may be used for collaborating on and sharing information and schedules, and Delicious or Diigo may be used for sharing web resources. These software programs and procedures facilitate cooperative endeavors from any geographical location and at any time of day. Skype allows multiple users to engage in a computer-assisted video conference call. TypeWithMe, TitanPad, and other programs allow group members to create a joint document. Google Calendar enables group members to plan when their group will meet both face-to-face and on-line. Students can engage in multiuser games with players in many different geographic sites. There are so many tools for communicating among students and facilitating cooperation being developed that it is difficult to keep up with them. The expansion of network infrastructure and bandwidth, furthermore, have made cooperation more feasible than ever. Students can now cooperate through the web with other students in their group, school, and across the globe. The web also enables students to interact with subject experts. One of the most exciting, successful, and well-known web cooperative projects is the JASON project (www.jasonproject.org). This project focuses on engaging students in grades 4–9 in hands-on scientific discovery. With the help of multimedia tools and Internet broadcasting technology, participating students become part of a virtual research community. Among other things, they can accompany real researchers in real time as they explore polar regions, oceans, volcanoes, rainforests, and almost any other region of the world. In addition to the advances in software, tools such as the iPad are increasingly making technology-assisted cooperative learning more feasible, effective, involving, and fun.

## WebQuests

WebQuests organize cooperative groups to complete inquiry-oriented projects among students so they can learn about a particular problem or area of interest. Students can be from the same class or from different geographic locations. Students are expected to use the information they are learning to complete a practical task while engaging in such activities as analysis, synthesis, and evaluation. Examples of WebQuests may be found at WebQuest Taskonomy.

## Creating a website

Cooperation may be enhanced through having each cooperative group build a web page or website reflecting the nature of the group, its goals and purposes, and the progress the group is making toward achieving its goals. Webpages and websites can help create, clarify, and institutionalize the group's identity. A group portfolio may be included that contains the best work of each member relevant to the group goals. There are a number of tools to help build websites, such as Facebook, Google Sites, Blackboard Engage, or wikis.

## Web-enabled multiplayer simulation games

What makes web-enabled, multiplayer, simulation games interesting is that while the interface, surroundings, characters, situations, and challenges are simulated, players actually interact with each other. They tend to facilitate cooperative endeavors to solve problems in an enjoyable, engaging way. Examples of multiplayer simulation games are Civilization (e.g., it allows players to match wits against history's greatest leaders as they strive to build and rule an empire) and The Sims (e.g., players' avatars interact as they go about daily life). Such games allow students to communicate with students in other schools and countries. Doing so may broaden their perspectives and enable them to learn about other cultures, languages, and issues. Students may supplement the game playing with emails, such as ePALS.

## Shared bookmarking

Computers make it increasingly easier to track the work of students and cooperative groups through social bookmarking sites. Cooperative groups can also set up their own social bookmarking sites. Google Bookmarks and Diigo are examples of social bookmarking sites.

## Course management

Learning communities may be created for students through web-enabled course management systems (CMS) programs. CMS programs allow teachers securely to post information, share resources, and encourage online discussions. Moodle, Blackboard Academic Suite and Goodle Apps for Education are examples.

## Summary and conclusions

Technology does not have to isolate and separate students. When used effectively, technology can bring students together in cooperative efforts and enhance student experiences. Accessing information through the internet can broaden the curriculum deepen students' learning. Instructional technology can remove geographical and communication barriers that limit learning. Technology can provide students with immediate feedback. By enabling students to cooperate in learning to read, write, and discuss, work with several medias simultaneously, illustrate reports, create multimedia projects, cover relevant events together, create websites and webpages, engage in inquiry projects that take place in any corner of the world, and play multiplayer simulation games requiring them to solve problems and live together peacefully, technology can revolutionize how members of cooperative groups interact and work other. Teachers can use technology to track the work of students and cooperative groups and create learning communities both within the classroom and the world as a whole. None of these uses of technology has to separate or isolate students, instead these technological tools can enable and enhance the cooperative learning experiences of students.

### Conflict of interest statement

The authors declare that the research was conducted in the absence of any commercial or financial relationships that could be construed as a potential conflict of interest.
